# Supplementary material for: Gliosarcoma Is Driven by Alterations in PI3K/Akt, RAS/MAPK Pathways and Characterized by Collagen Gene Expression Signature
Source: Cancers (Basel). 2019 Feb 27;11(3):284. doi: 10.3390/cancers11030284 (PMC6468745; doi:10.3390/cancers11030284)
Supplement: Supplementary file 1 [file cancers-11-00284-s001.pdf]

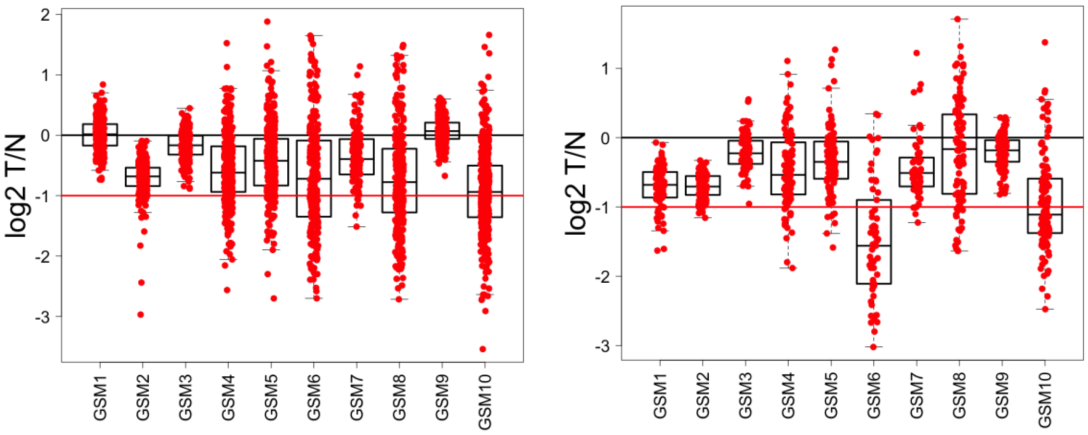

Figure S1. Focal deletion in a gene locus of *NF1* (left panel) and *PTEN* (right panel) genes.

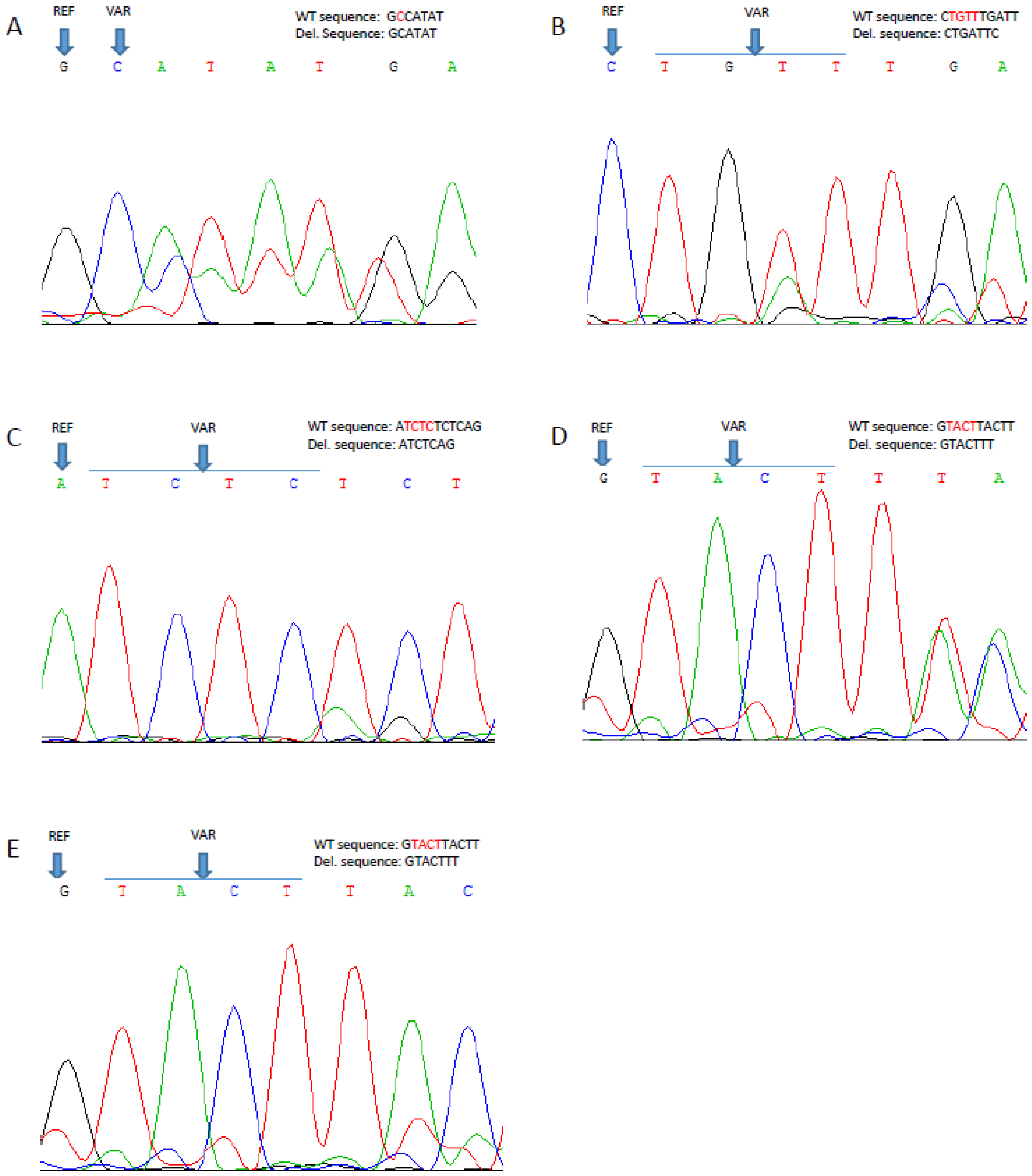

Figure S2. Validation of *NF1* and *PTEN* deletions detected using Sanger sequencing. (**A**) *NF1*, pos. 17:31325895, ref:G, var: -C, sample GSM3. (**B**) *NF1*, pos. 17:31235638, ref:C, var: -TGTT, sample

GSM4. (**C**) *NF1*, pos. 17:31159044, ref:A, var: -TCTC, sample GSM9. (**D**) *PTEN*, pos. 10:87961041, ref:G, var: -TACT, sample GSM3. (E) *PTEN*, pos. 10:87961041, ref:G, var: -TACT, sample GSM4.

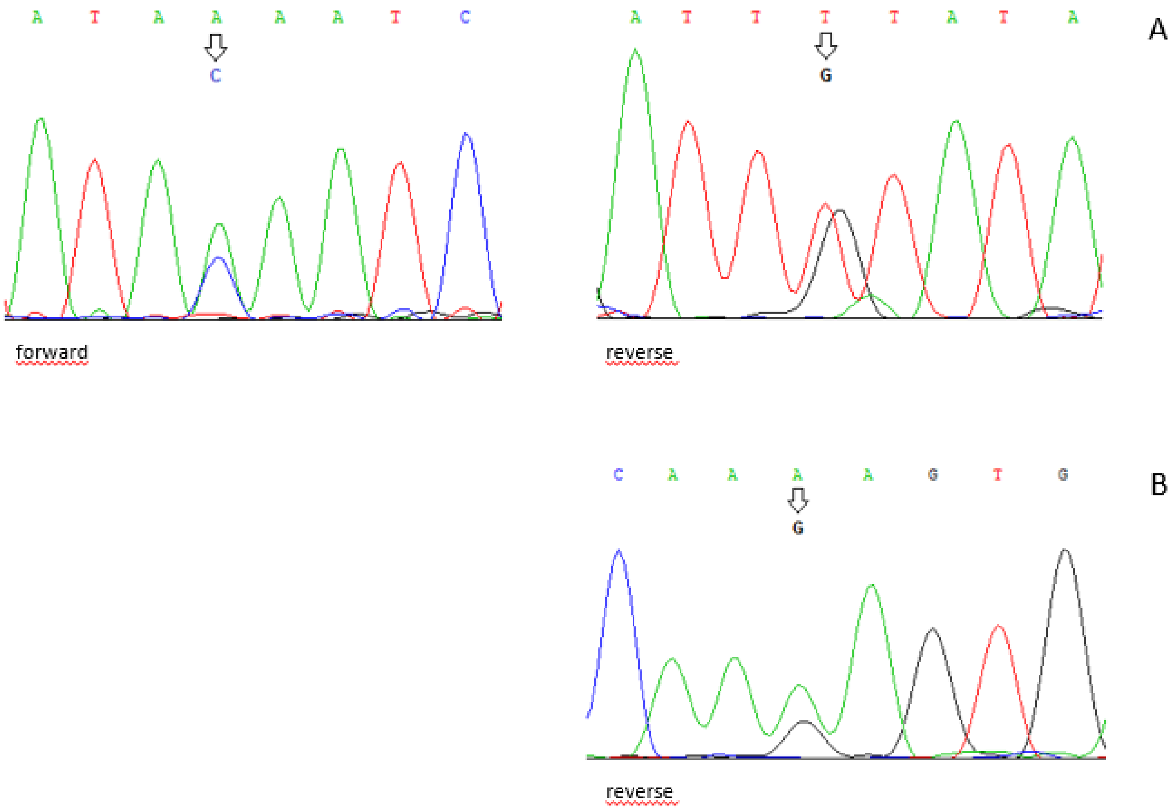

Figure S3. Validation of single nucleotide variants detected using Sanger sequencing. (**A**) *PTEN*, pos. 10:87925552, ref:A, var:C, sample GSM2. (**B**) *PTEN*, pos. 10:87960910, ref:T, var:C, sample GSM9. No representative result was obtained with forward primer due to presence of deletion in the immediate vicinity of tested variant.

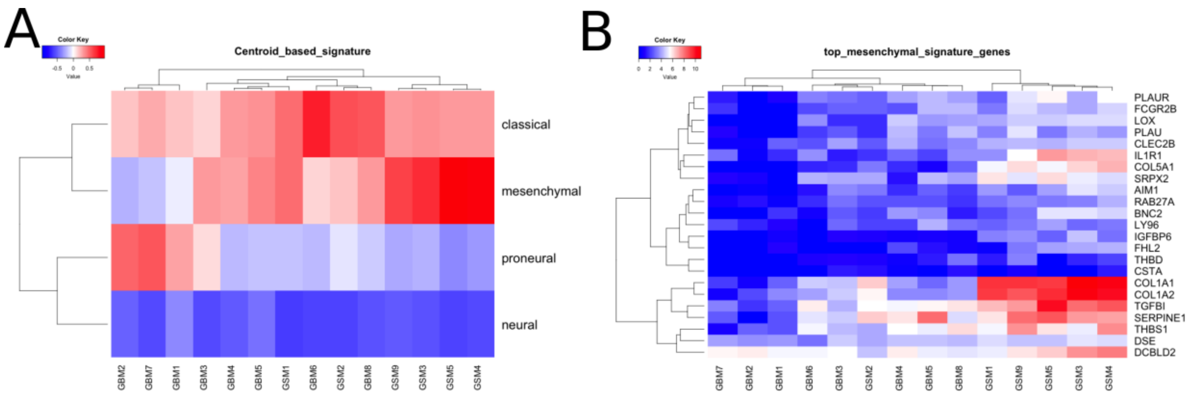

Figure S4. Gliosarcoma data comparison to glioblastoma subtypes. (**A**) RNA-seq profiles of gliosarcoma (GSM) and glioblastoma (GBM) samples were assigned to four TCGA subtypes based on the centroid based signature (https://tcga-data.nci.nih.gov/docs/publications/gbm_exp/). (**B**) Heatmap of GSM and GBM samples gene expression, only top genes important for a mesenchymal subtype are presented.

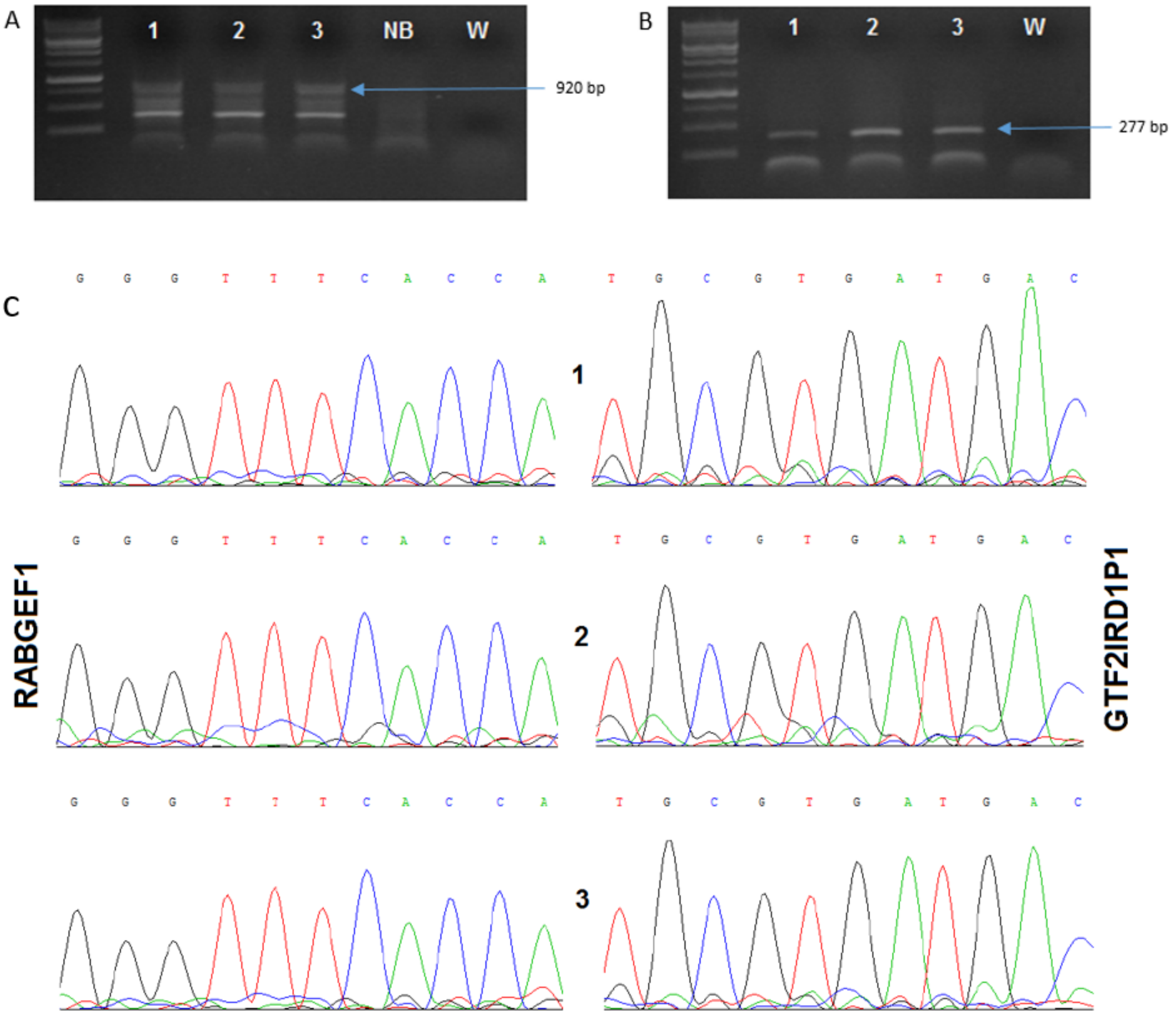

Figure S5. Validation of chromosome translocation *RABGEF1—GTF2IRD1P1* in 3 gliosarcoma samples using a NESTED PCR method. (**A**) Agarose gel electrophoresis results obtained after PCR with primers for a long sequence product (920 bp). Mix of products after the analysis was obtained due to high similarity to other regions in genome. (**B**) Agarose gel electrophoresis results obtained after PCR with primers for a short sequence product (277 bp). (**C**) Final PCR product obtained after NESTED PCR (277 bp) was sequenced using Sanger sequencing. Proper sequences of both genes (RABGEF1 –NCBI Reference Sequence: NC_000007.13; GTF2IRD1P1—NCBI Reference Sequence: NC_000007.13) were found on the opposite side of the product. 1—GSM1, 2—GSM3, 3—GSM9, NB—normal brain DNA sample, W—water control. A 1 kb marker ladder was loaded to estimate molecular size.

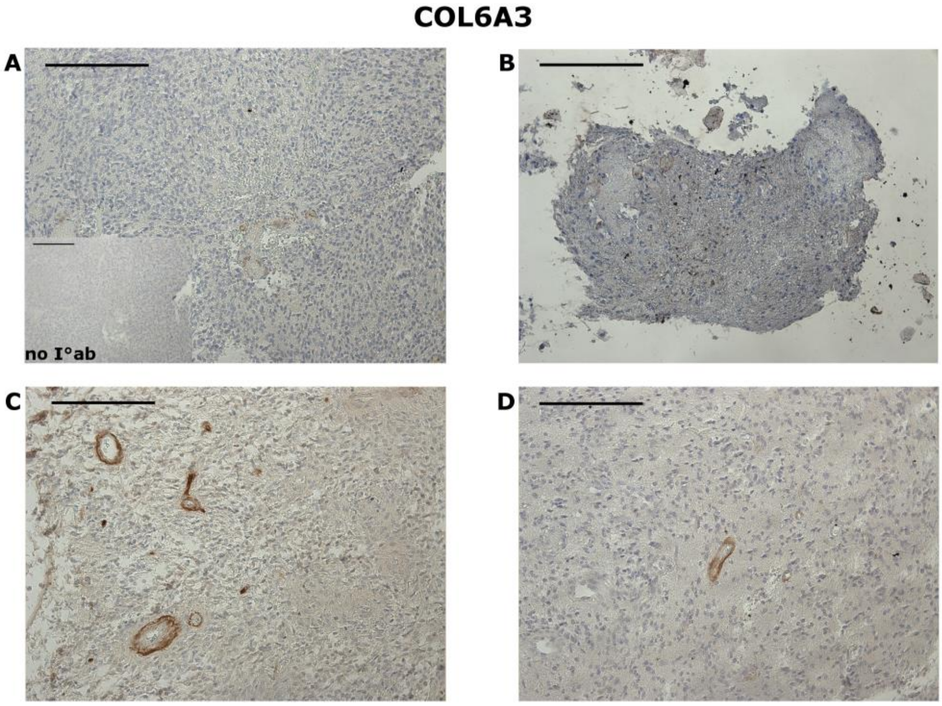

Figure S6. The expression of collagen 6A3 in glioblastoma tissue sections. The positive COL6A3 staining is present only in blood vessels across all sections from different patients (**A**–**D**). Bars: 200 μm.

Table S1. Patients clinical information.

number	Age at diagnosis (y)	Sex	Tumor location	Primary/Secondary	Survival [m]	Therapy
GSM1	60	f	frontal right	primary	8	Radiotherapy
GSM2	73	m	temporal left	primary	3	Radiotherapy
GSM3	44	m	tempo-parietal left	primary	6	Radiotherapy + Temodal
GSM4 *	60	m	occipital, left, containing local bleeding	primary	5.3	
GSM5	66	m	right temporal	primary	8.8	
GSM6	50	m	right frontal intercranial/intraparenchymatous	primary	8.8	
GSM7 **	52	f	right parieto-occipital	secondary	14.2	
GSM8	81	m	right frontal	primary	2.3	
GSM9	82	f	temporal right	primary	0	nothing
GSM10	26	f	left temporal	primary	10	

f—female, m—male, *—Clinical history: Treatment for thyroid carcinoma, **—Clinical history: GBM diagnosed in 2008.

Table S2. Estimated tumor purity.

Samples	Mean alpha
GSM1	0.4675
GSM2	0.61
GSM3	0.511
GSM4	0.423
GSM5	0.35
GSM6	0.473
GSM7	0.436

GSM8	0.72
GSM9	0.357
GSM10	0.54

Supplementary Materials and Methods

Tumor Samples

Glioblastoma (GBM) samples were obtained from the Canadian Brain Tissue Bank or were collected at the Institute of Psychiatry and Neurology. Sample collection was conducted under the protocol #14/KBE/2012 and approved by the institutional review board of The Institute of Psychiatry and Neurology, Warsaw. All samples were classified according to the WHO criteria for CNS tumors.

DNA/RNA Isolation

Total DNA and RNA were extracted from fresh frozen GSM tissue samples using Trizol Reagent (Thermo Fischer Scientific, Waltham, MA USA), following the manufacturer's protocol. RNA after Trizol extraction was stored in -80°C. To obtain high quality genomic DNA, Trizol isolated DNA was incubated overnight with proteinase K (600 μg/mL) to remove protein contamination and an equal volume of phenol-chloroform-isoamyl alcohol mixture was added. After centrifugation the top aqueous phase was carefully transferred to a new tube, extracted with an equal volume of chloroform and centrifuged again. The top aqueous phase was precipitated with two volumes 100% ethanol in the presence of 250 mM NaCl in −20 °C. Then, after centrifugation the DNA pellet was washed with 70% ethanol and dried at room temperature and resuspended in small amounts of H_2O. RNA quality was estimated using the RNA 6000 Nano assay on the Agilent Bioanalyzer (Agilent Technologies, Santa Clara, CA, USA). Samples with acceptable RIN ≥ 7 were used for further analyses. DNA from whole blood from GSM patients was isolated using Quick-gDNA MiniPrep kit from Zymo Research (Zymo Research Corp, Irvine, CA, USA), according to manufacturer's protocol. DNA was quantified with a Nanodrop (Thermo Fischer Scientific, Waltham, MA USA), and DNA purity assessed by the A260/A280 and A260/A230 absorbance ratios.

DNA Sequencing

DNA isolated from tumor samples was processed for library preparation according to a NimbleGen SeqCap EZ Library SR (v 4.2) user guide. Briefly, 1 μg of gDNA was sheared to a mean size of 300 bp in a 50 μL volume using a Covaris microTUBE screwcap and the Covaris M220 system according to the standard protocol. The sheared sample was prepared into a library using the KAPA Library Preparation Kit for Illumina platforms (KAPABiosystems, KR0935 v2.14) according to the manufacturer's instructions. The final libraries were run on the Agilent BioAnalyzer 2100 using the DNA High Sensitivity Kit (Agilent; 5067-4626) and Quantus Fluorometer (Promega, Madison, WI, USA) to assess the size distribution and library molarity. Libraries were then prepared for Illumina cluster generation and sequencing. Paired-end sequencing, resulting in 76 bases from each end of the fragments, was performed using a HiSeq 1500 Genome Analyzer (Illumina, San Diego, CA, USA). Tumor samples were sequenced at the enriched regions with a planned mean coverage of 100×, meaning on average, each base of our targeted enrichment panel will be covered 100 times. DNA isolated from whole blood samples was sequenced with a mean coverage of 30×. DNAs from blood samples were used to verify a somatic status of mutations detected in tumor samples as well as in the CNV analysis.

Identified mutations were verified using Sanger sequencing. Fragments of genomic DNA were amplified using polymerase chain reaction (PCR) with specific primers (primer sequences are presented in the Table S3) and Taq polymerase (EURx Ltd., Gdansk, Poland) using Eppendorf Mastercycler Nexus. All PCR primers were designed using Primer3 to generate PCR products of 300–500 bp. The presence of PCR product of the proper size was confirmed using 1% agarose gel

electrophoresis. Sanger sequencing of PCR products was performed at the Oligo.pl sequencing facility.

Sequence Alignment Pipeline

In the process of analysis, all reads contaminated by sequencing adapter sequences, as well as reads that in a bin of 20 bp reads had mean quality below Q30, were discarded. A minimal length of a read used for mapping was set as 75 bp.

Validation of chromosome translocation RABGEF1–GTF2IRD1P1 was performed using the NESTED PCR method using starters designed in our laboratory, according to optimized parameters of reaction—as seen in Table S3 below. For PCR Hot Start Taq polymerase (Roche, Basel, Switzerland) and Eppendorf Mastercycler Nexus (Eppendorf, Hamburg, Germany) were used. The presence of the PCR product of the proper size was detected using agarose gel electrophoresis.

Primer sequences, annealing temperature, number of cycles and concentration of Mg^{2+} used in NESTED-PCR assay.

Table S3. Primer sequences.

	**Sequence (5′–3′)**	**Tm/cycles/Mg^{2+}**
RABGEF1–GTF2IRD1P1 (long)	F: GATCAGAGGTCTGTGTGCCA<br>R: ACTGAGTCAGCTGGTTCCCA	53^0C/35/1,5mM
RABGEF1–GTF2IRD1P1 (short)	F: ACAGAAGTTCATGAGTTTGTTTTGT<br>R: ACATCTGTGTCATCACGCACT	52^0C/35/1,5mM

Protein Atlas

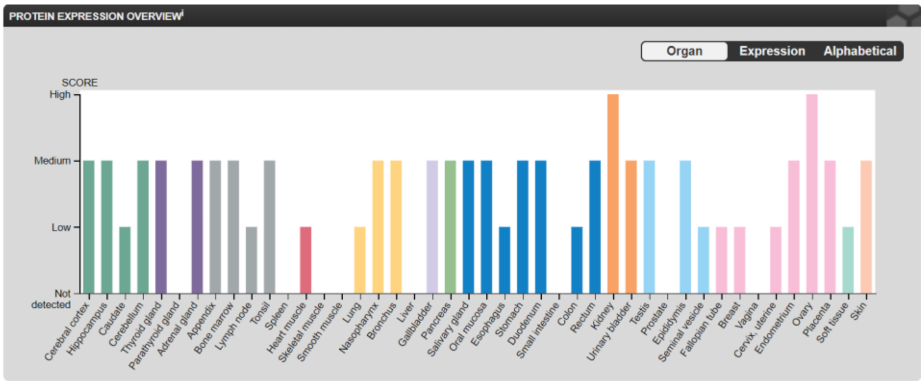

COL1A1

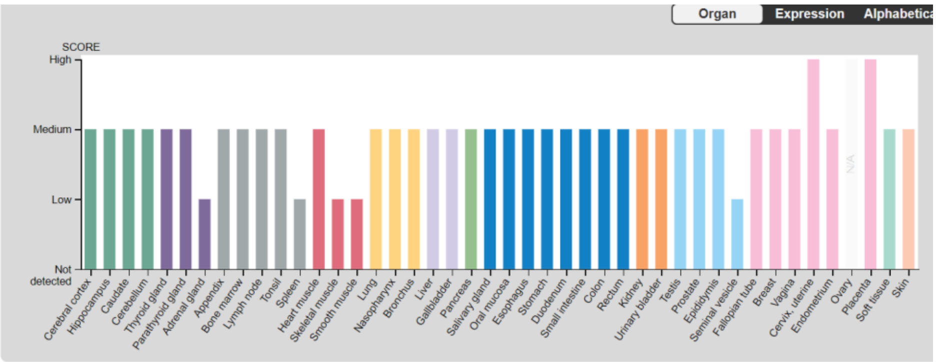

COL1A2

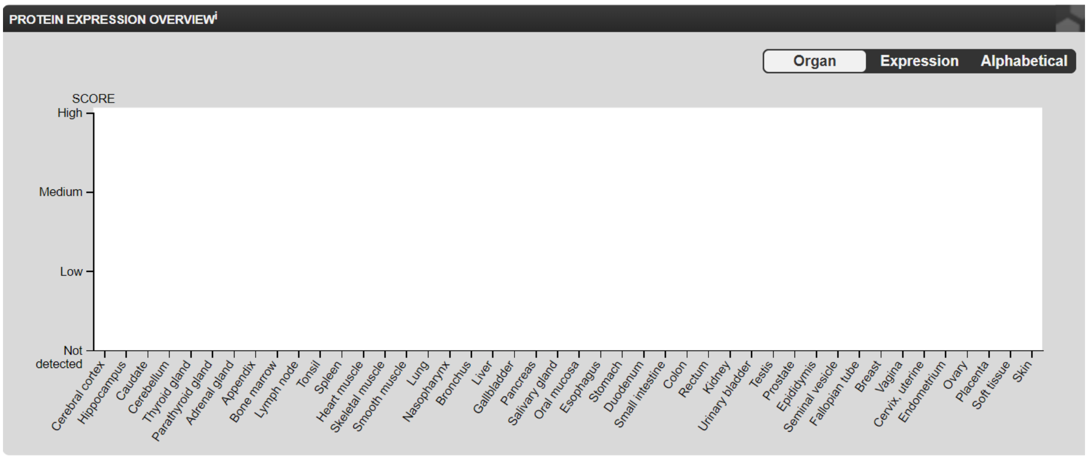

COL3A1

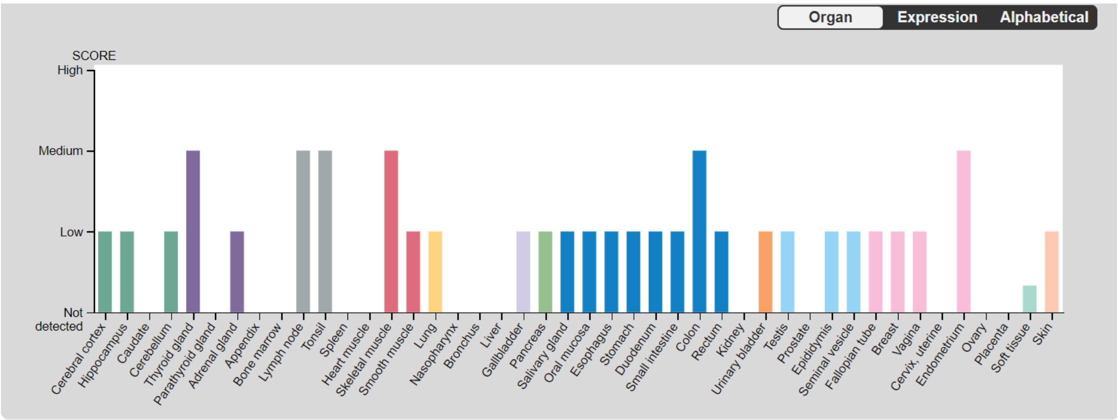

COL5A1

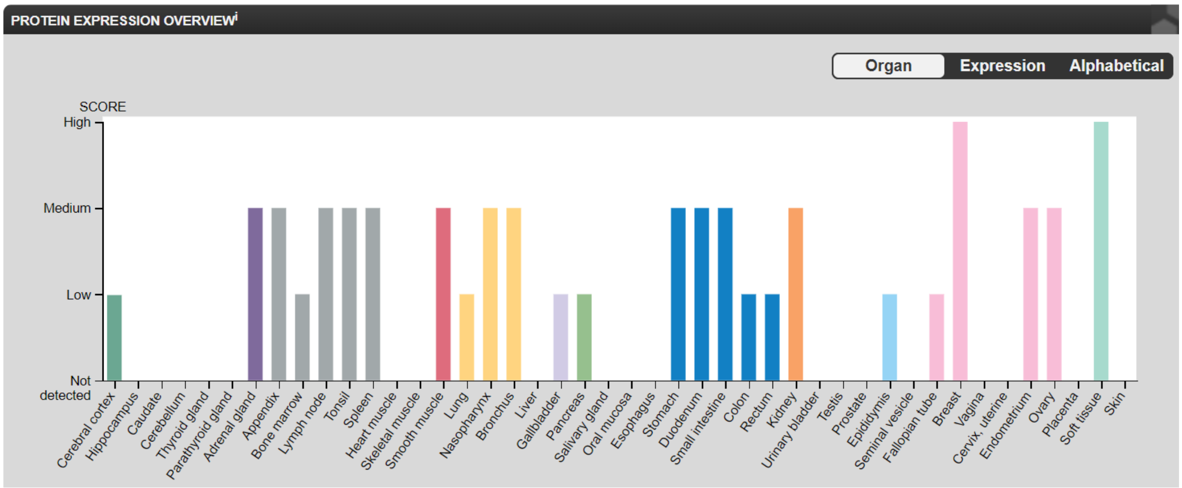

COL6A2

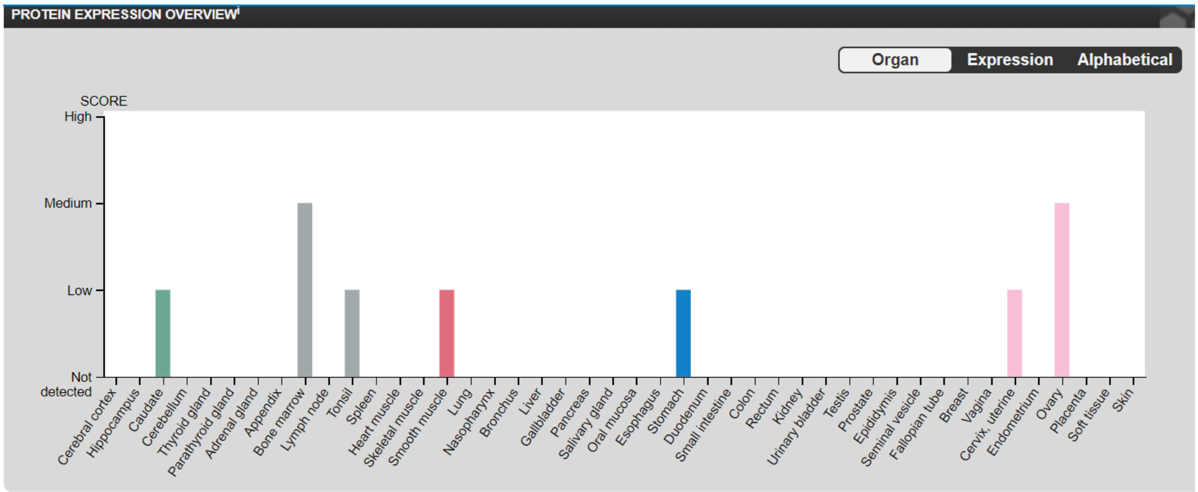

COL6A3

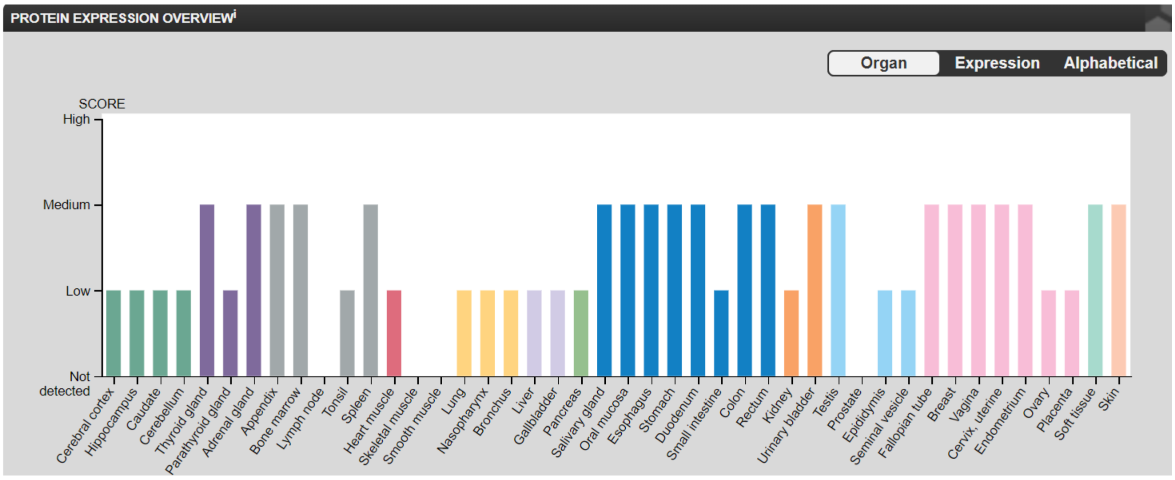

COL18A1
